# Anticancer Potential of Fisetin Against Glioblastoma: In Vitro Evaluation, Radiostability Assessment, and Preliminary PLGA Encapsulation

**DOI:** 10.3390/polym17223074

**Published:** 2025-11-20

**Authors:** Agnieszka Sobczak, Katarzyna Dominiak, Bartłomiej Sztenc, Barbara Jadach, Aneta Woźniak-Braszak, Mikołaj Baranowski, Paweł Bilski, Aleksandra Majchrzak-Celińska, Violetta Krajka-Kuźniak, Anna Jelińska, Maciej Stawny, Aleksandra Gostyńska-Stawna

**Affiliations:** 1 Poznan University of Medical Sciences, Chair and Department of Pharmaceutical Chemistry, 3 Rokietnicka, 60-802 Poznań, Poland; asobczak@ump.edu.pl (A.S.); katarzyna.dominiak@student.ump.edu.pl (K.D.); 79471@student.ump.edu.pl (B.S.); ajelinsk@ump.edu.pl (A.J.); 2 Poznan University of Medical Sciences, Doctoral School, 70 Bukowska, 60-812 Poznań, Poland; 3 Poznan University of Medical Sciences, Chair and Department of Pharmaceutical Technology, 3 Rokietnicka, 60-802 Poznań, Poland; bajadach@ump.edu.pl; 4 Adam Mickiewicz University, Functional Materials Physics Division, Faculty of Physics and Astronomy, 2 Uniwersytetu Poznańskiego, 61-614 Poznań, Poland; aneta.wozniak-braszak@amu.edu.pl (A.W.-B.); mikolaj.baranowski@amu.edu.pl (M.B.); 5 Adam Mickiewicz University, Department of Experimental Physics of Condensed Phase, Faculty of Physics and Astronomy, 2 Uniwersytetu Poznańskiego, 61-614 Poznań, Poland; pawel.bilski@amu.edu.pl; 6 Poznan University of Medical Sciences, Chair and Department of Pharmaceutical Biochemistry, 3 Rokietnicka, 60-802 Poznań, Poland; majchrzakcelinska@ump.edu.pl (A.M.-C.); vkrajka@ump.edu.pl (V.K.-K.)

**Keywords:** PLGA nanoparticles, radiation sterilization, NMR, EPR, cytotoxicity, glioblastoma

## Abstract

(1) Background: Glioblastoma is the most common and aggressive primary brain tumor in adults, with a median survival time for patients treated with standard chemotherapy often of less than 1 year. Potential anticancer activity against glioblastoma is demonstrated by flavonoids, including fisetin (FIS). Although, its clinical application is limited by poor solubility and chemical instability. This study aimed to conduct a preliminary evaluation of fisetin’s suitability for intravenous delivery by developing and characterizing FIS-loaded poly(lactic-co-glycolic acid) nanoparticles (FIS-PLGA-NPs) and assessing their in vitro cytotoxic potential against glioblastoma. (2) Methods: Six FIS-PLGA nanoparticle formulations were prepared via the emulsification–solvent evaporation method and evaluated for key physicochemical properties. The biological activity of fisetin was examined through cell cycle analysis and apoptosis assays, and the most promising formulation was further assessed using an MTT assay in U-138 MG glioblastoma cells. In parallel, pure fisetin was exposed to ionizing radiation, including the standard sterilization dose of 25 kGy, to evaluate its structural stability and suitability for terminal sterilization approaches. (3) Results: The selected formulation (NP4) exhibited a mean particle size of approximately 330 nm, a zeta potential of −7.2 mV, a polydispersity index of 0.25, and high encapsulation efficiency and drug loading of 83.58% and 13.93%, respectively. Despite its preliminary nature, this formulation retained cytotoxic activity in vitro. Moreover, pure fisetin maintained its structural and chemical integrity following radiation exposure, supporting the feasibility of radiation sterilization prior to nanoparticle incorporation. (4) Conclusions: These findings confirm the feasibility of combining radiosterilizable fisetin with PLGA-based nanoencapsulation and provide an initial foundation for the development of an injectable fisetin delivery system for glioblastoma treatment. Further optimization, particularly surface modification, will be required to enhance colloidal stability and systemic performance.

## 1. Introduction

Fisetin (3,3′,4′,7-tetrahydroxyflavone, FIS) is a natural flavonoid widely distributed in various fruits and vegetables, such as strawberries, apples, and onions, as well as in wine and tea. It has been regarded as having a broad spectrum of potential health-promoting properties. FIS is primarily known for its antioxidant, anti-inflammatory, neuroprotective, anticancer, senolytic, and cardioprotective properties. Its anticancer activity has been observed in liver, lung, cervix, prostate, bladder, leukemia, and glioma models [1,2,3].

FIS can induce apoptosis through the activation of both exogenous (death receptor) and endogenous (mitochondrial) pathways, which is associated with the activation of caspases-3, -8, and -9, as well as an increased expression of the pro-apoptotic protein BAX, and reducing the levels of anti-apoptotic proteins such as Bcl-2. This compound also negatively affects the MAPK, PI3K, and HIF-α pathway, essential in resistance in B cells [4,5,6].

The anticancer potential of FIS against glioblastoma has also been confirmed in numerous in vitro and in vivo models. Its mechanism of action involves multiple molecular targets and signaling pathways, leading to modulation of oxidative stress and DNA damage (genotoxic effects), which consequently inhibit the proliferation and induce apoptosis of cancer cells [4]. It has also been proven in glioma cells that FIS reduces their invasiveness and migration, among other mechanisms, by inhibiting the expression of the ADAM9 metalloproteinase and activating the ERK1/2 pathway [7,8]. Moreover, FIS exhibits direct cytotoxic effects on glioma cells and indirect effects by modulating the tumor microenvironment. Specifically, Joma et al. [9] demonstrated that FIS differentially modulates oxidative stress and activates pathways related to autophagy and antioxidant response, including Nrf2–KEAP1 and TFEB, depending on the cell type. TFEB activation and increased expression of antioxidant genes were observed in microglial cells, whereas these effects were significantly weaker in glioma cells. This suggests a potential selective protective action toward non-cancerous cells while exerting a pro-apoptotic impact in transformed cells [9].

As previously mentioned, FIS can act either by directly inhibiting tumor cells at high concentrations or as an adjuvant in combination therapy. Preclinical studies have shown that its combination with conventional chemotherapeutics, such as temozolomide, doxorubicin, or cisplatin, can enhance treatment efficacy through synergistic effects, reducing cell resistance to treatment and limiting systemic toxicity [4,8,10,11]. It was also shown that FIS could alleviate cardiotoxicity caused by doxorubicin [12]. In combination therapies, complex nanoformulations are being explored to enhance the bioavailability of FIS, improve its ability to cross the blood–brain barrier, and increase its affinity for tumor cells. Examples include FIS-loaded liposomes combined with cisplatin or micelles co-delivered with doxorubicin [10,11].

An essential mechanism of FIS action, particularly in the context of glioblastoma, is its senolytic activity. Cancer cells treated with temozolomide often undergo senescence and exhibit a senescence-associated secretory phenotype (SASP), which can promote tumor progression and resistance to treatment. FIS selectively eliminates senescent cells, reducing their viability and secretion of pro-inflammatory cytokines [8].

Despite promising in vitro and in vivo studies, no active clinical trials currently assess the efficacy of fisetin in glioblastoma patients. One Early Phase I trial (NCT07025226) has been registered on ClinicalTrials.gov, which involves sequential or combination administration of dasatinib, quercetin, FIS, and/or temozolomide to patients with previously treated glioma with residual disease; however, as of the preparation of this manuscript, recruitment has not yet begun.

Based on the available data, FIS appears to be a promising candidate for adjuvant glioma treatment. However, several factors currently limit the therapeutic effectiveness of FIS, including its lipophilic nature, which leads to poor targeting, low chemical stability, and limited bioavailability [1,4]. Its poor aqueous solubility (<1 mg/mL) also hinders intravenous administration, despite this route offering 100% bioavailability. Consequently, developing novel drug delivery systems to overcome these limitations is critically essential [13,14]. FIS nanoformulations currently under investigation for cancer applications include, among others: nanoemulsions and liposomes (e.g., in lung carcinoma) as well as polymeric micelles (e.g., in ovarian, breast, colon cancer models) [1,4].

This study aimed to explore the anticancer potential of fisetin against glioblastoma through a comprehensive in vitro evaluation of its cytotoxic, cell cycle-modulating, and pro-apoptotic effects in U-138 MG cells. In parallel, a preliminary PLGA-based nanoencapsulation approach was undertaken to investigate the feasibility of using biodegradable nanoparticles as a future delivery platform for intravenous administration. Furthermore, the radiostability of pure fisetin was assessed following exposure to ionizing radiation to determine its suitability for such sterilization, which is essential for the development of sterile pharmaceutical formulations. Together, these investigations provide an initial foundation for the advancement of fisetin toward injectable nanoformulations intended for glioblastoma treatment.

## 2. Materials and Methods

### 2.1. Materials

FIS was sourced from Pol-Aura (Olsztyn, Poland). Poly(D, L-lactide-co-glycolide) (PLGA, Resomer RG 502, 50:50) and polyvinyl alcohol (PVA) were purchased from Sigma-Aldrich (St. Louis, MO, USA). All organic solvents used in the study were of analytical or high-performance liquid chromatographic (HPLC) grade.

### 2.2. Cell Culture

The U-138 MG cell line (ATCC-HTB-16) was acquired from the American Type Culture Collection (ATCC; Manassas, VA, USA). Cells were cultured in Eagle’s Minimum Essential Medium (EMEM; Merck, Darmstadt, Germany) supplemented with fetal bovine serum (FBS; Biowest, Nuaillé, France) and penicillin–streptomycin (Merck, Darmstadt, Germany). The cultures were kept at 37 °C in a humidified incubator (Memmert, Schwabach, Germany) under an atmosphere of 5% CO_2_ and 95% air.

#### 2.2.1. Cell Cycle Distribution

Cell cycle distribution was assessed using the Muse^®^ Cell Cycle Kit (Merck, Darmstadt, Germany) following the manufacturer’s instructions. In brief, cells (3 × 10^5^ per well) were seeded into 6-well plates and allowed to adhere for 24 h before treatment. Afterwards, the tested compounds were applied, and cultures were incubated for an additional 24 h. Cells exposed to 100 nM topotecan (TOPO) served as a positive control for inducing cell cycle arrest. Following treatment, cells were detached by trypsinization, rinsed with PBS, fixed in ice-cold 70% ethanol, and stored at −20 °C until staining. After overnight fixation, cells were washed again with chilled PBS, stained with propidium iodide in the presence of RNase A, and incubated for 30 min at room temperature in the dark. Fluorescence was then measured using the Muse^®^ Cell Analyzer, and the resulting data were processed with the Muse^®^ 1.5 Analysis Software.

#### 2.2.2. Apoptosis Analysis

The exposure of phosphatidylserine on the outer leaflet of the plasma membrane—an established hallmark of apoptosis—was assessed using Annexin V staining with the Muse^®^ Annexin V & Dead Cell Kit (Merck, Darmstadt, Germany), following the provided protocol. The incorporation of 7-Aminoactinomycin D (7-AAD) enabled differentiation between early and late apoptotic populations. To perform the assay, cells (3 × 10^5^ per well) were seeded into 6-well plates and allowed to adhere for 24 h before compound treatment. After the test substances were added, cultures were incubated for another 24 h. Cells exposed to 100 nM TOPO served as a positive reference for apoptosis induction.

After incubation, cells were detached using trypsin, stained with Annexin V and 7-AAD, and kept at room temperature in the dark for 20 min. Samples were then subjected to flow cytometric analysis using the Muse^®^ Cell Analyzer, and the resulting data were processed with Muse^®^ 1.5 Analysis Software.

### 2.3. Preparation Process of Fisetin-Loaded PLGA Nanoparticles

PLGA nanoparticles loaded with FIS (FIS-PLGA-NPs) were prepared using the emulsification–solvent-evaporation method. Six formulations (FIS-PLGA-NP1 to FIS-PLGA-NP6) were developed to investigate the influence of FIS-to-PLGA ratio, PVA concentration, and sonication time on nanoparticle properties (Table 1). FIS (approximately 2.5 and 10.0 mg) was accurately weighed into 5 mL glass vials, dissolved in 1.0 mL of methanol to obtain 2.5 and 10.0 mg/mL solutions. Separately, PLGA (approximately 50.0 mg) was dissolved in 1.0 mL of dichloromethane and added to the FIS solutions. To prepare PVA stabilizing solutions, 5.0, 10.0, and 15.0 g of PVA were dissolved in distilled water (final volume 500.0 mL) at 65 °C with stirring at 550 rpm, yielding 1%, 2%, and 3% (*w*/*v*) PVA solutions.

Organic phase (combined PLGA and FIS solutions) was added to 30 mL of aqueous PVA solution (1%, 2%, or 3%) in 50 mL beakers. Emulsification was carried out in an ice-water bath using a Sonopuls HD 2070 ultrasonic homogenizer (Bandelin Electronic GmbH & Co. KG, Berlin, Germany) (40% amplitude, 30 s on/off cycles) for 2, 4, or 6 min. The emulsion was then stirred at 350 rpm for 24 h at room temperature to evaporate the organic solvent. The resulting suspensions were washed three times with distilled water and centrifuged (FrontierTM 5000 Multi Pro FC5718R centrifuge, Ohaus, NJ, USA) at 5500 rpm for 30 min to remove residual PVA and unencapsulated FIS. All received suspensions of microparticles were placed in glass vials, filled to a height approx. 2 cm, frozen (−40 °C; 24 h) and then subjected to lyophilization. Process was prepared in Christ Epsilon 2-4 LSC plus (Osterode am Harz, Germany) with conditions −40 °C; 0.2 mbar, time 56 h. Dried materials were stored in the 4 °C for further investigation.

### 2.4. Characterization of Developed Fisetin-Loaded PLGA Nanoparticles

#### 2.4.1. Mean Particle Size (MPS), Polydispersity Index (PDI), and Zeta Potential (ZP) Analysis

The mean particle size (MPS), polydispersity index (PDI), and zeta potential (ZP) of the FIS-PLGA-NPs were measured using a Malvern Zetasizer Nano ZS (Malvern Instruments, Malvern, UK). Dynamic light scattering (DLS) and electrophoretic light scattering (ELS) techniques were employed to determine MPS and ZP, respectively. The lyophilizate of each formulation was dissolved in water (0.5 mg of lyophilizate/mL of water). Subsequently, 100 µL of the obtained solutions was diluted with deionized water to a final volume of 10 mL and transferred into dedicated polycarbonate cuvettes for MPS, PDI, and ZP analysis.

#### 2.4.2. Drug Loading (DL%) and Entrapment Efficiency (EE%)

DL% and EE% were determined using UV-Vis spectrophotometric analysis using a Lambda 20 spectrophotometer (PerkinElmer). Samples were prepared by accurately weighing approximately 5.0 mg of lyophilized nanoparticles into 10.0 mL volumetric flasks, filling to volume with distilled water, and sonicating for 5 min. A 2.0 mL aliquot of this solution was mixed with 0.5 mL of methanol and diluted to 10.0 mL with acetonitrile. The absorbance of the resulting solution was measured at 360 nm. FIS content was calculated based on the calibration curve parameters.

The DL% and EE% were calculated using the following equations [15]:

(1) $$DL\%=\frac{m_{FIS}}{m_{FIS-PLGA-NPs}}\times 100\%,$$

where $m_{FIS}$ is the mass of fisetin incorporated, and $m_{FIS-PLGA-NPs}$ is the mass of lyophilized fisetin-loaded PLGA-based nanoparticles;

(2) $$EE\%=\frac{{DL\%}_{actual}}{{DL\%}_{teoretical}}\times 100\%,$$

where ${DL\%}_{actual}$ is the actual drug loading and ${DL\%}_{teoretical}$ is the theoretical drug loading.

#### 2.4.3. The Effect of Developed Fisetin-Loaded PLGA Nanoparticles on Cell Viability

In order to analyze the cytotoxicity of FIS-PLGA-NPs, the MTT test was performed. Briefly, the cells (10,000 cells per well) were seeded on the 96-well plates and left for 24 h. Afterwards, the analyzed compounds in the concentration range of 1 µM up to 100 µM were added to the cells and left for 24 h of incubation. DMSO-treated cells served as controls. After the time mentioned above, the cells were washed twice with warm PBS and incubated for 3 h in the presence of a fresh medium containing MTT salt (0.5 mg/mL). Then the formazan crystals were dissolved in acidic isopropanol. The absorbance was measured at 570 and 690 nm on a Tecan Infinite M200 microplate reader (Grödig, Austria).

#### 2.4.4. In Vitro Drug Release Studies

The dialysis bag diffusion method was employed to investigate the release of FIS from lyophilized FIS-PLGA-NPs. One milliliter of the lyophilizate dispersion in water, equivalent to 1 mg of FIS, was placed into a pre-swelled dialysis bag (molecular weight cutoff: 14,000 Da) and immersed in 100 mL of 70% ethanol, which served as the release medium. The system was maintained under stirring at 200 rpm and 37 °C. Samples (1 mL) were withdrawn at predetermined time intervals and replaced with an equal volume of fresh medium to maintain sink conditions. The release study was conducted in triplicate. Quantitative analysis of the released FIS was performed by HPLC using the standard solution (10 μg FIS/mL).

### 2.5. Development of the Fisetin Sterilization Method

Approximately 0.5 g of FIS was placed in a colorless glass of 5 mL volume, and the glass was closed with a plastic stopper. The samples were exposed to irradiation in a linear electron accelerator LAE 13/9 with an electron beam of 9.96 MeV and a current intensity of 6.2 mA until they absorbed a 25, 100, and 400 kGy dose.

#### 2.5.1. Electron Paramagnetic Resonance (EPR) Spectroscopy

EPR measurements were made on an Adani X-band spectrometer using the eSpinoza control software v1.0.35.2. The spectrometer working parameters are presented in Table 2. Non-irradiated and irradiated FIS were weighed and placed in thin-walled glass tubes with a diameter of 5 mm. A standard tetracyanoquinodimethane (TCNQ) sample with an exceptionally narrow linewidth was used and securely fixed to prevent any positional changes. This approach enabled comparison of individual spectra relative to the cavity side without the need to account for the quality factor (Q) of the resonant cavity in the analysis. During data processing, individual spectra were scaled to match the amplitude of the reference spectrum, after which the reference spectrum was subtracted.

#### 2.5.2. Fourier Transform Infrared (FT-IR) Spectroscopy

FT-IR spectra were collected on an IRAffinity-IS Fourier Transform Infrared Spectrophotometer (Shimadzu, Kyoto, Japan) instrument in the range of 4000–400 cm^−1^, with a resolution of 4.0 cm^−1^ and 40 scans. For tablet preparation, 1 mg of unirradiated or irradiated FIS was weighed and combined with 300 mg of KBr, and then the IR spectra were recorded.

#### 2.5.3. Nuclear Magnetic Resonance (NMR) Analysis

Solid-state ^1^H NMR measurements of the spin-lattice relaxation times T_1_ in the laboratory frame were carried out on a pulse spectrometer operated at 25 MHz (El-Lab Tel-Atomic, Poznań, Poland) [16]. The spin-lattice relaxation times (T_1_) were measured using the saturation recovery pulse sequence within the temperature range of 80 K to 300 K. The T_1_ values were determined by fitting a single-exponential function, Mz(t) = M_0_(1 − exp(−t/T_1_)), to the magnetization recovery of the magnetization (Mz) as a function of time (t), where M_0_ denotes the equilibrium magnetization. Measurement errors were kept below 10% for all data.

#### 2.5.4. High-Performance Liquid Chromatography (HPLC)

FIS was determined in samples collected before and after irradiation using an HPLC method specifically developed and validated for this study. Method optimization included selecting mobile phase composition (qualitative and quantitative assessment of phosphoric acid, acetic acid, water, acetonitrile, and methanol), stationary phase (Poroshell 120 EC-C18 150 × 3.0 mm, 2.7 µm, Luna RP-C18 150 × 4.66 mm, 5 µm), mobile phase flow rate, and column temperature. The final method employed a reversed-phase C18(2) column (100 Å Luna, 150 × 4.6 mm, 5 µm; Phenomenex, Torrance, CA, USA). A gradient elution using 0.1% phosphoric acid (A) and acetonitrile (B) was applied at a flow rate of 1.0 mL/min. The gradient program was as follows: 0–11 min: 25→70% B, 11–12 min: 70→25% B, 12–15 min: 25% B. Gradient elution was selected to enhance the detection of potential degradation products from irradiation. Analyses were performed on an Agilent 1260 Infinity II LC system (Agilent Technologies, Böblingen, Germany) equipped with a quaternary pump, in-line degasser, vial sampler (maintained at 25 ± 0.8 °C), column oven (35 ± 0.8 °C), and a diode-array detector (DAD) set at 360 nm.

The developed analytical method was validated by the guidelines of the International Council for Harmonisation (ICH Q2(R2)) [17]. Stock solutions of FIS (~0.2 mg/mL) were prepared by dissolving the compound in methanol and then diluted across the calibration range (n = 9) for validation. Precision and accuracy were assessed using samples at a concentration of ~20 μg/mL, prepared in nine replicates. Test samples were obtained by dissolving 10 mg of irradiated FIS in 10 mL of methanol (n = 3), followed by dilution to yield final concentrations of approximately ~20 μg/mL (n = 9) and ~100 μg/mL (n = 3). The higher concentration was explicitly used to assess the potential presence of degradation products. Based on calibration curves, results were calculated as mean values ± absolute error.

The method demonstrated excellent linearity over the range of 2.58–145 μg/mL, with a regression equation of y = 38.887 ± 0.230x and a correlation coefficient of r = 0.99998. The detection (LOD) and quantification (LOQ) limits were determined to be 0.65 μg/mL and 1.97 μg/mL, respectively. Precision and accuracy, both intra-day and inter-day, were assessed at the ~20 μg/mL level, based on two independent series comprising 9 replicates each (total n = 18). The method exhibited satisfactory precision, as indicated by the relative standard deviation (RSD) values in Table 3. Furthermore, the method was accurate, with recovery rates ranging from 96.87% to 102.57% (Table 3).

## 3. Results and Discussion

### 3.1. Biological Activity of Fisetin

According to the literature data, FIS exerts cytotoxic effects on various types of glioma and glioblastoma cell lines, including LN299 [8], U-87 MG [10], lipopolysaccharide (LPS)/interferon gamma (IFNγ) activated C6 glioma cells [18], T98G and BEAS-2B cells [5]. These findings prompted us to further investigate the anticancer activity of FIS in another glioblastoma model, namely the human U-138 MG cell line. To gain deeper insight into the underlying mechanisms of its cytotoxic action, we conducted cell cycle analysis and apoptosis assay following FIS treatment.

#### 3.1.1. Effect of Fisetin on Cell Cycle Distribution in U-138 MG Glioblastoma Cells

**Figure 1:**
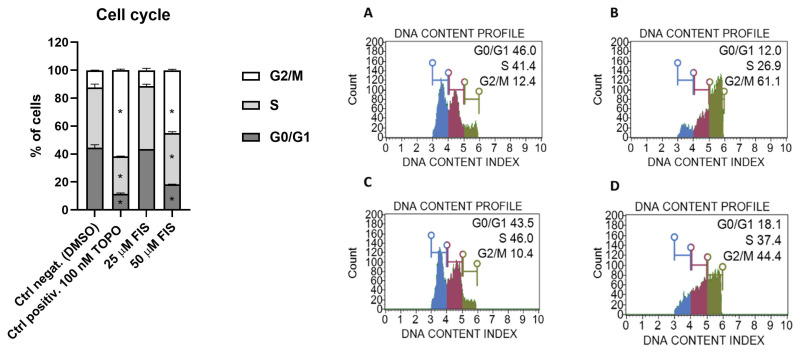
The influence of fisetin (FIS) on cell cycle distribution in the U-138 MG cell line ((**A**)—negative control (DMSO); (**B**)—positive control (TOPO); (**C**)—FIS 25 µM; (**D**)—FIS 50 µM). Asterisk indicates the value that is significantly different from the DMSO-treated control (*p* < 0.05 was considered statistically significant). Representative histograms are also presented, with blue indicating G0/G1 phases, violet S phase, and green G2/M phases.

The impact of FIS on the cell cycle progression of U-138 MG glioblastoma cells was evaluated by flow cytometric analysis after treatment with two concentrations of FIS (25 µM and 50 µM). The control group treated with DMSO exhibited a typical distribution of cell cycle phases. 25 µM FIS did not induce statistically significant changes in the distribution of cell cycle phases; however, the treatment with 50 µM FIS resulted in a pronounced cell cycle arrest (similar to that observed after 100 nM topotecan), suggesting a stronger cell cycle-blocking effect at the higher concentration. The obtained results are presented in Figure 1.

These observations support the potential of FIS to modulate glioblastoma cell proliferation by interfering with cell cycle progression.

#### 3.1.2. Effect of Fisetin on Apoptosis in U-138 MG Glioma Cells

**Figure 2:**
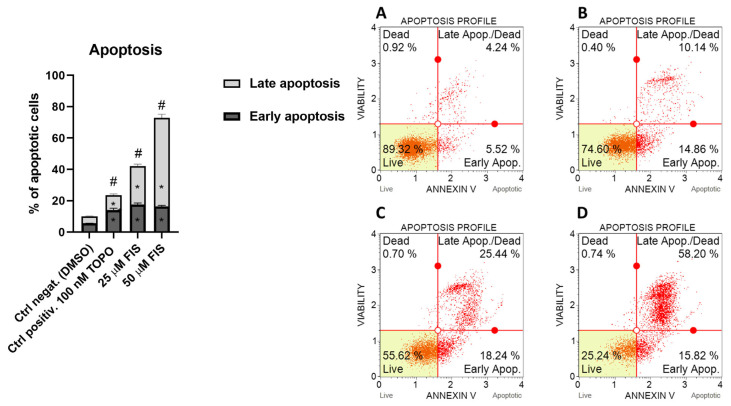
The impact of FIS on apoptosis in the U-138 MG cell line after 24 h of treatment ((**A**)—negative control (DMSO); (**B**)—positive control (TOPO); (**C**)—FIS 25 µM; (**D**)—FIS 50 µM). The asterisk (*) indicates statistically significant difference as compared to the DMSO-treated control for early/late apoptosis. Hashtag (#) above bar indicates statistically significant differences as compared to the DMSO-treated control for total apoptotic cells (*p* < 0.05 was considered statistically significant).

To investigate the pro-apoptotic activity of FIS, U-138 MG glioblastoma cells were treated with two concentrations of FIS (25 µM and 50 µM), and apoptosis was assessed. The control group treated with DMSO exhibited a low basal apoptosis level of 10%. Treatment with 25 µM FIS significantly increased total apoptosis to 42.05 ± 2.30%, while 50 µM FIS further enhanced the apoptotic response to 73%. The pro-apoptotic effect of FIS was concentration-dependent and markedly higher than that of the positive control with topotecan, for which the percentage of total apoptotic cells at 100 nM was 23.55 ± 2.05%. These results, presented in Figure 2, indicate that FIS effectively induces apoptosis in U-138 MG glioma cells, strongly impacting cell cycle regulation and cell survival pathways.

The observed apoptosis-inducing activity of FIS highlights its potential role as a chemotherapeutic or adjuvant agent targeting glioblastoma cells by promoting programmed cell death.

### 3.2. Characterization of Fisetin-Loaded PLGA Nanoparticles

Although FIS showed promising anticancer activity, its clinical application is limited by poor water solubility and low bioavailability. Therefore, in this study, we developed FIS-loaded PLGA nanoparticles (FIS-PLGA-NPs) to enhance the therapeutic potential of this agent against glioblastoma. Six different formulations were prepared to assess how variations in FIS-to-PLGA ratio, PVA concentration, and sonication time affect the physicochemical characteristics of the nanoparticles, as well as drug loading and entrapment efficiency (Table 4).

Among the developed formulations with varying FIS-to-PLGA ratios, FIS-PLGA-NP3 (FIS-to-PLGA ratio: 1:20) and FIS-PLGA-NP4 (FIS-to-PLGA ratio: 1:5) emerged as the most favorable in terms of physicochemical properties. These nanoparticles were prepared using a PVA concentration of 2% and a sonication time of 4 min, which proved to be optimal parameters for obtaining small particle sizes (323 ± 2 nm and 330 ± 6 nm, respectively), low polydispersity (≤0.25), and satisfactory EE%, particularly in FIS-PLGA-NP4, which had higher FIS content. Significantly, increasing the sonication time to 6 min and the PVA concentration to 3%, as shown in FIS-PLGA-NP6, further reduced the particle size, reaching the smallest diameter (300 ± 4 nm); however, such modification of the process negatively affected the DL% and EE%. The same approach was less effective at a lower FIS-to-PLGA ratio (FIS-PLGA-NP5), where particle size remained relatively large (380 ± 7 nm). These results showed that although more intense emulsification and higher PVA concentration lead to reduced particle size in the case of a high FIS-to-PLGA ratio (FIS-PLGA-NP6), likely due to improved dispersion of FIS within the polymer matrix. However, this also results in lower EE%, possibly due to increased drug diffusion into the aqueous phase during nanoparticle formation. In contrast, FIS-PLGA-NP1 and NP2, which were prepared with lower PVA concentration (1%) and shorter sonication time (2 min), showed significantly larger particle sizes (660 ± 80 nm and 560 ± 21 nm, respectively) and higher PDI values (>0.45), suggesting suboptimal emulsification and a broader size distribution. Such characteristics may negatively affect nanoparticle stability and cellular uptake. All formulations were characterized by a negative zeta potential, ranging from −13.60 mV (FIS-PLGA-NP1) to −6.92 mV (FIS-PLGA-NP6), with more negative values observed for formulations characterized by a lower FIS-to-PLGA ratio under each set of formulation conditions. Taken together, formulation conditions II combined with a FIS-to-PLGA ratio of 1:5 resulted in the most optimal nanoparticle formulation (FIS-PLGA-NP4). Such an approach appears to offer the most favorable physicochemical profiles, with uniform particle sizes, acceptable surface charge characteristics, and satisfactory DL% and EE%. The obtained size range (≤330 nm) is consistent with values reported in the literature for PLGA-based nanoparticles developed for glioblastoma therapy [15,19], suggesting potential for further functionalization to enhance endocytosis-mediated or receptor-mediated cellular uptake and penetration into the tumor microenvironment [20,21].

While the relatively large size of PLGA nanoparticles (~330 nm) developed in the presented study may raise concerns regarding the ability of diffusion across the BBB, it is important to emphasize that, despite improved brain delivery observed for particles <100 nm, sizes in the range of 100–345 nm do not eliminate the ability of nanoparticles to reach the central nervous system. In vivo pharmacokinetic data from Gao and Jiang [22] clearly demonstrate measurable active substance levels in cerebrospinal fluid, cerebrum, and cerebellum following intravenous administration of nanoparticles within this size range. Although polysorbate-coated nanoparticles of 70 nm achieved higher brain exposure (e.g., cerebellum C_max = 82.03 ng/g), larger nanoparticles (63.49–66.35 ng/g) still resulted in significant drug accumulation within brain tissue, indicating a quantitative rather than qualitative difference in BBB permeation. These findings suggest that while surface-coated, ultra-small nanoparticles maximize BBB transport, nanoparticles of 100–345 nm retain the capacity to cross the barrier to a clinically relevant extent.

Previous studies on fisetin-loaded PLGA nanoparticles were conducted exclusively for oral delivery, demonstrating that such formulations can enhance fisetin’s solubility and protect it from premature degradation [23,24,25,26]. These formulations were obtained through nanoprecipitation or double emulsion techniques [23,24,25,26], sometimes in combination with solubility enhancers such as cyclodextrins [25]. In contrast, the present study did not aim to replicate or surpass those findings, but rather to conduct a preliminary evaluation of the feasibility of developing fisetin-loaded PLGA nanoparticles for potential intravenous administration and verify their cytotoxic effect on glioblastoma cell lines, which have never been tested yet.

Within this context, the formulation FIS-PLGA-NP4 achieved an encapsulation efficiency of 83.58%, which is comparable to, and in some cases slightly higher than, values reported in previous fisetin-based PLGA systems (typically 70–80%) [23,24,25,26]. Although the particle size obtained here (330 nm) is larger than those commonly described in oral studies and may appear less favorable from a pharmacokinetic perspective, drug loading was intentionally prioritized at this stage to ensure adequate payload for subsequent functionalization and targeting strategies relevant to systemic delivery, such as in glioma therapy. Unlike some earlier approaches, no complexing agents or co-polymers were employed, meaning that fisetin was incorporated directly into the PLGA matrix, providing a basis for controlled release without excipient interference.

**Figure 3:**
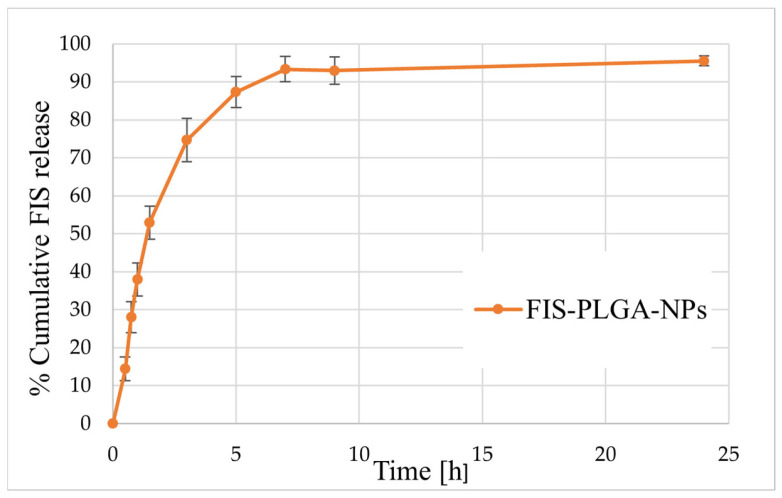
The release profile of FIS from FIS-PLGA-NP4 in 70% ethanol at 37 °C (mean ± SD, n = 3).

Given these formulation characteristics, subsequent studies focused on evaluating the release behavior of FIS-PLGA-NP4 under accelerated conditions to gain insight into its diffusion and matrix performance. Such testing is often employed to obtain preliminary information on release kinetics within a shorter timeframe. According to the literature, accelerated release may be achieved by modifying factors such as temperature, pH, or by adding surfactants and organic solvents to the release medium, which can enhance drug solubility and polymer permeability [27,28]. In this study, a 70% ethanol solution was used as the release medium, ensuring complete solubility and stability of fisetin (FIS) while simulating accelerated conditions consistent with the “worst-case scenario” concept. Under these conditions, over 70% of FIS was released from the PLGA matrix within three hours, with the release approaching a plateau of 93–95% after approximately seven hours (Figure 3). Previous studies have shown that organic solvents such as acetonitrile or ethanol can markedly accelerate drug release from PLGA matrices by increasing polymer porosity and hydration without inducing chemical degradation of the polymer. Therefore, a release medium containing a high concentration of ethanol could serve as an effective tool for accelerated testing, enabling a rapid evaluation of the formulation’s behavior and the structural response of the carrier under extreme conditions [27]. However, such conditions should be interpreted with caution, as the strong solubilizing effect of ethanol and the plasticization of PLGA may not fully represent the release behavior under physiological conditions. Prior to selecting this medium, preliminary test were conducted with commonly used media (PBS, PBS containing 20% ethanol, and PBS with Tween 80), which under our experimental conditions appeared less suitable for maintaining full sink conditions, likely due to partial precipitation and limited stability of FIS.

### 3.3. Biological Activity of FIS-Loaded PLGA NPs

**Figure 4:**
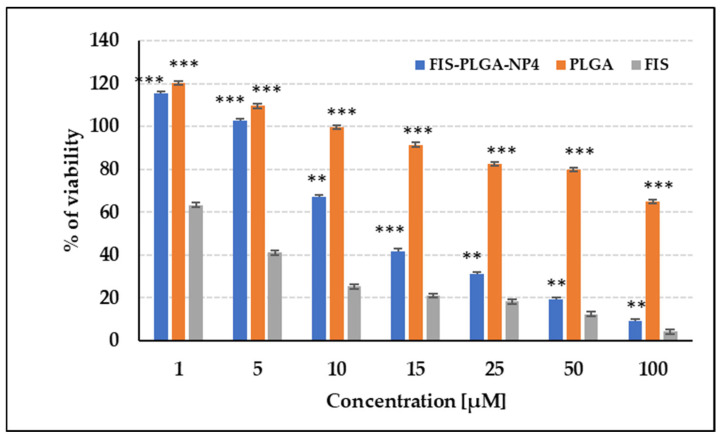
The impact of FIS on the viability of the U-138 MG cell line in the MTT test. Mean results are shown ± SEM. Statistically significant differences between the effects of PLGA or FIS-PLGA-NP4 versus FIS are denoted with an asterisks (** *t*-test, *p* ≤ 0.01; *** *t*-test, *p* ≤ 0.001).

In the MTT assay performed on U-138 MG glioblastoma cells, free FIS exhibited considerable activity on cells, indicating the effectiveness of this compound in reducing cell viability. The most optimal formulation of FIS (FIS-PLGA-NP4) demonstrated strong, dose-dependent cytotoxic activity (Figure 4). A significant reduction in cell viability was already evident at 25 µM (to approximately 31%), with viability further decreasing to 9–19% at higher concentrations (50–100 µM), confirming improved efficacy of the nanoparticle formulation. Importantly, empty PLGA nanoparticles (PLGA-NP) showed no cytotoxicity across the entire concentration range, confirming that the observed effect was solely attributable to the presence of FIS and not the carrier itself. These results indicate that the FIS-PLGA-NP4 formulation is characterized by satisfactory anticancer activity against glioblastoma cells. Collectively, these findings highlight the therapeutic potential of PLGA nanoparticles as an effective delivery system for FIS in glioblastoma treatment.

### 3.4. Evaluation of the Impact of Radiation Sterilization on Fisetin Stability

Ensuring the suitability of fisetin for pharmaceutical applications requires verification of its stability under sterilization conditions. A comprehensive analysis was conducted to assess the physicochemical stability of FIS upon exposure to ionizing radiation, including visual examination, EPR spectroscopy, HPLC, FT-IR spectroscopy, and NMR relaxometry. These studies aimed to determine whether ionizing radiation, routinely applied at a sterilization dose of 25 kGy, affects the chemical structure or leads to the formation of free radicals in FIS. Additionally, higher radiation doses (up to 400 kGy) were applied for exploratory purposes to enable a more in-depth investigation of the effects of radiation on the compound’s physicochemical properties. NMR-based analysis of molecular dynamics was performed exclusively for the sample irradiated with the highest dose (400 kGy) to optimize the detection of subtle structural or dynamic changes.

#### 3.4.1. Visual Examination

FIS, a yellow-orange, odorless crystalline powder, showed no visible changes in appearance or odor after exposure to ionizing radiation doses ranging from 25 to 400 kGy. Although no macroscopic signs of degradation were observed, advanced analytical techniques were employed to detect potential structural changes at the molecular level. In most studies assessing the effects of sterilization, observable changes, particularly color shifts from white to yellow, are commonly reported as indicators of the substance degradation or transformation [29]. In the case of FIS, which is inherently yellow, such visual changes may be masked. Therefore, relying solely on visual inspection is insufficient, and further in-depth analytical methods are necessary to detect potential chemical alterations. Such methodological rigor aligns with best pharmaceutical practice [29,30].

#### 3.4.2. EPR Analysis

**Figure 5:**
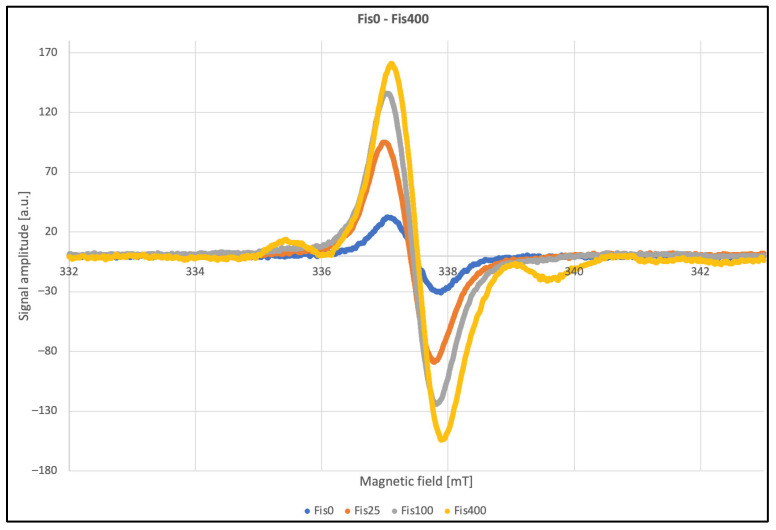
EPR spectra of FIS before and after irradiation.

To ensure accurate analysis of the spectral parameters, measurements were performed under various modulation settings, with careful consideration of clipping effects on linewidth and signal quality. Overmodulation leads to artificial line broadening; however, relative signal intensities remain comparable. The corresponding EPR spectra are shown in Figure 5. The linewidth values provided in the table below illustrate the impact of second-harmonic modulation, making these parameters unreliable for precise analysis. To obtain accurate linewidth measurements (Table 5), data were acquired using a modulation amplitude approximately half the linewidth. No significant differences were observed under these conditions, and the average linewidth was determined to be 4.3 G. Although theoretical guidelines recommend using modulation amplitudes up to eight times smaller than the linewidth, this approach was not feasible due to the low signal intensity. To further validate these results, a multiharmonic analysis was performed. This method involves detecting multiple harmonics of the modulated signal, which allows more comprehensive characterization of the spectral line shape and reduces the impact of modulation-induced distortions. By comparing the relative amplitudes of higher harmonics, it was possible to confirm that the measured linewidths were consistent with those obtained under optimized conditions. Additionally, multiharmonic analysis allows for the accurate determination of spectral parameters regardless of the second harmonic modulation amplitude, even if substantial overmodulation does not pose a problem. An additional advantage of this technique is the improved signal-to-noise ratio [31].

**Figure 6:**
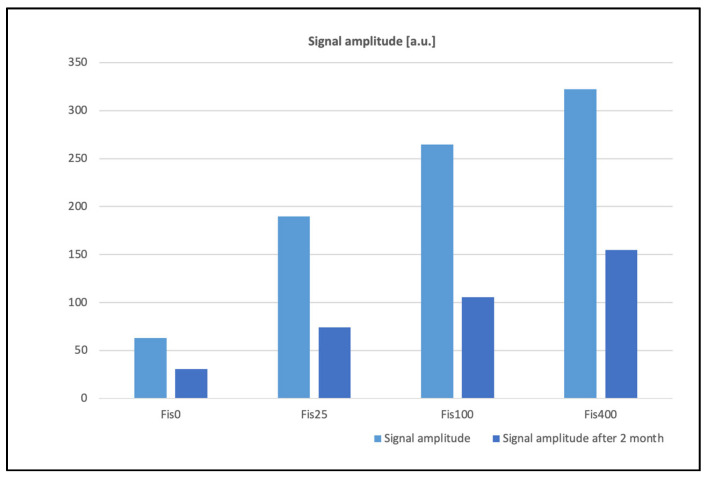
EPR signals amplitudes for FIS.

Significant changes in EPR signal amplitudes were observed as a function of the absorbed dose of ionizing radiation. The induced paramagnetic centers gradually decay over time; after approximately two months, signal amplitudes decreased by more than half, as shown in Figure 6. These effects can occur due to several processes related to the nature and stability of radiation-induced defects. Paramagnetic centers created by ionizing radiation, such as free radicals or sites with unpaired electrons, may gradually recombine with ions or other lattice defects, leading to a decrease in EPR signal intensity. Additionally, irradiation often generates vacancies and interstitial atoms, which can migrate at room temperature and annihilate each other, reducing the number of stable paramagnetic centers. Chemical reactions, such as oxidation or interaction with residual oxygen or moisture in the sample, may also contribute to signal decay over time. Some paramagnetic centers are metastable and can undergo spontaneous relaxation to a diamagnetic state due to structural rearrangements or spin-lattice interactions, further diminishing the observed EPR signal. Other spectral parameters remained stable within the limits of experimental uncertainty.

#### 3.4.3. HPLC, FT-IR, and NMR Analysis

**Figure 7:**
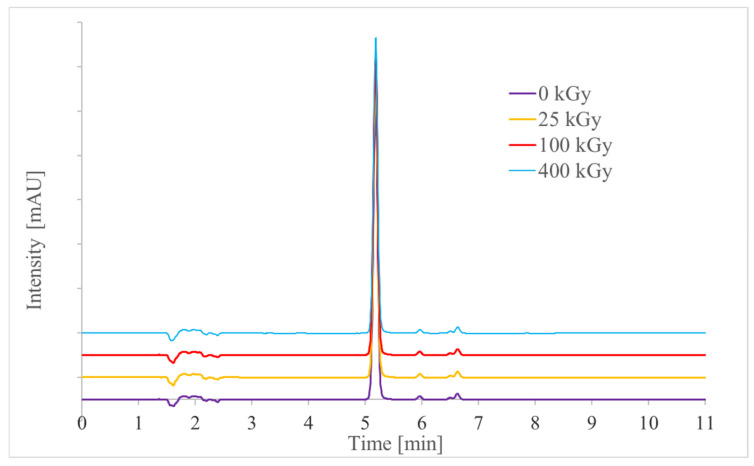
Chromatograms of samples before and after radiation (doses of 25–400 kGy) of FIS; the FIS concentrations were about 20 μg/mL.

To evaluate the chemical stability of FIS after exposure to ionizing radiation, a dedicated HPLC method was developed and validated. Following irradiation with doses ranging from 25 to 400 kGy, a slight decrease in FIS content was observed compared to the non-irradiated control sample (ranging from 1.52% to 2.59%). This minor reduction may indicate a limited effect of irradiation or, more likely, result from the method’s variability (Table 6). Moreover, no additional peaks were detected in the chromatograms of irradiated samples, as assessed using a diode array detector (DAD). The peaks at retention times t_R_ = 5.9 min and 6.6 min appeared consistently in all chromatograms, including those of non-irradiated samples (Figure 7), suggesting they are not related to irradiation. These findings suggest that the tested compound exhibits considerable stability under exposure to ionizing radiation.

**Figure 8:**
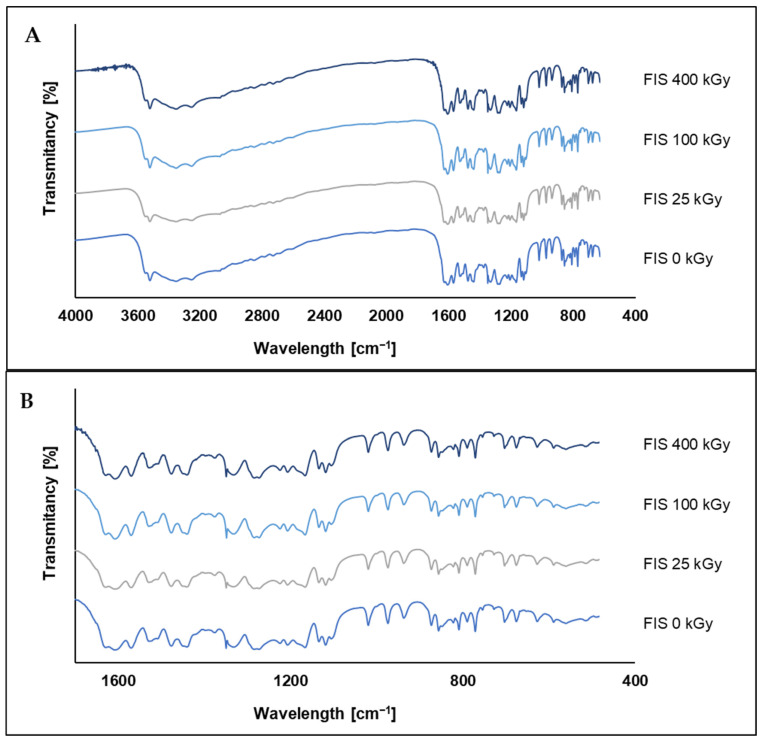
FT-IR spectra of FIS before and after irradiation: (**A**)—full range spectra; (**B**)—fingerprint range spectra.

FT-IR spectra of FIS irradiated with doses ranging from 25 to 400 kGy were compared to the spectrum of the non-irradiated sample (0 kGy) to assess potential structural changes induced by ionizing radiation (Figure 8). Such analysis indicates that the chemical structure of FIS remains largely unaffected, even at high radiation doses. In the region between 3600 and 3200 cm^−1^, which corresponds to the O–H stretching vibrations of hydroxyl groups, a broad band is observed consistently across all samples. Minor differences in intensity and width of this band are present but appear to be subtle and may reflect slight physicochemical variations or instrumental noise rather than significant degradation of hydroxyl functionalities. Similarly, in the region around 1700–1600 cm^−1^, associated with C=O and aromatic C=C stretching vibrations, the bands remain stable in both position and intensity, suggesting that the aromatic core of FIS is preserved under the applied radiation conditions.

In the fingerprint region (1600–400 cm^−1^), presented in Figure 8B, which includes C–O stretching, aromatic ring deformations, and other characteristic vibrational modes, no notable spectral changes are observed. The shape, number, and intensity of the bands remain virtually unchanged across all irradiation doses. The absence of new peaks, peak shifts, or loss of characteristic bands supports the conclusion that irradiation does not cause detectable chemical degradation of FIS, at least within the resolution limits of the FT-IR technique.

The FT-IR and HPLC analyses of FIS irradiated with a dose of 25 kGy revealed no significant structural or chemical changes. The FT-IR spectra remained virtually unchanged across all samples, with consistent band positions and intensities, indicating preservation of the main functional groups and the aromatic core of the molecule. Similarly, HPLC analysis did not show any degradation products or significant loss of FIS content at 25 kGy, confirming the chemical stability of FIS under sterilization conditions.

**Figure 9:**
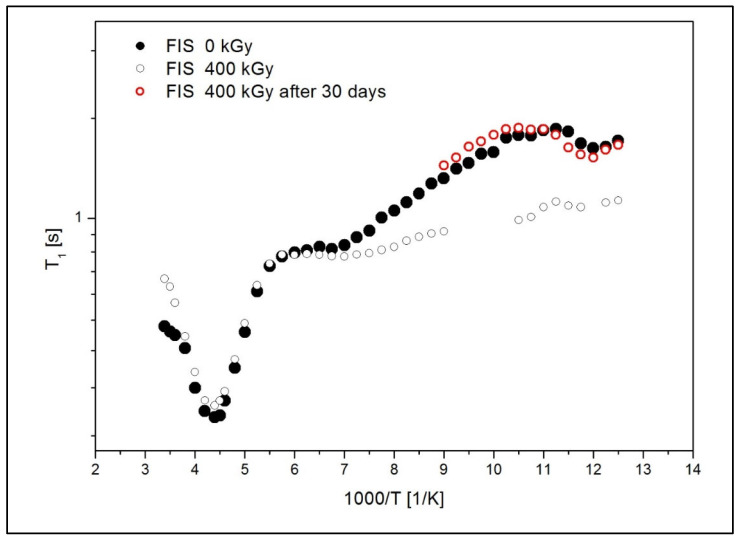
The temperature dependence of spin-relaxation time (T_1_) for FIS 0 kGy and FIS 400 kGy, respectively.

Given the absence of detectable alterations at the 25 kGy dose, relaxation times (T_1_) studied by NMR were conducted exclusively on the sample irradiated with the highest dose (400 kGy). This approach was chosen to maximize the likelihood of detecting potential structural or dynamic changes at the molecular level that may not be evident using less sensitive techniques such as FT-IR or HPLC. Figure 9 presents the temperature dependence of the spin-lattice relaxation times (T_1_) in the laboratory frame for the non-irradiated FIS 0 kGy and the irradiated FIS 400 kGy, respectively.

In the low-temperature range, a significant shortening of the T_1_ relaxation times was observed for the irradiated sample. This indicates that irradiation produces changes in the molecular dynamics and the structure of the drug. The faster relaxation observed in the irradiated sample may result from the dissociation of bonds present in pure FIS. It is also assumed that radiation contributes to the formation of free radicals in the system, which could further shorten the relaxation times. Notably, relaxation time measurements for FIS irradiated at 400 kGy were repeated after one month, showing relaxation time T_1_ values comparable to those of the non-irradiated FIS 0 kGy sample. These results suggest that the molecular structure in the irradiated sample rebuilds over time.

The activation parameters describing the molecular dynamics of FIS samples with ionizing radiation of 0 kGy and 400 kGy were estimated by analyzing the temperature dependence of the spin-relaxation times T_1_ described by Bloembergen–Purcell–Pound (BPP) theory [32]. The T_1_ values depend on dipolar interactions modulated by the internal motions of the molecule. Since these contributions are additive, the relaxation rate of the multi-proton system can be obtained from the following expression [32,33]:

(3) $$\frac{1}{T_{1}}=\frac{2}{3}\gamma^{2}\sum ∆M_{2k}\left(\frac{\tau_{ck}}{1+\omega_{0}^{2}\tau_{ck}^{2}}+\frac{4\tau_{ck}}{1+{4\omega }_{0}^{2}\tau_{ck}^{2}}\right),$$

where γ is the gyromagnetic ratio of protons, ΔM_2k_, is the reduction in the second moment in ^1^H NMR spectra induced by the contributing motion, and τ_0_ is the correlation time expressed by the Arrhenius formula:

(4) $$\tau_{ck}=\tau_{0}exp\left(\frac{E_{ak}}{RT}\right),$$

where τ_0_ is the pre-exponential factor, E_ak_ is the activation energy of molecular motion, and R is the gas constant.

**Figure 10:**
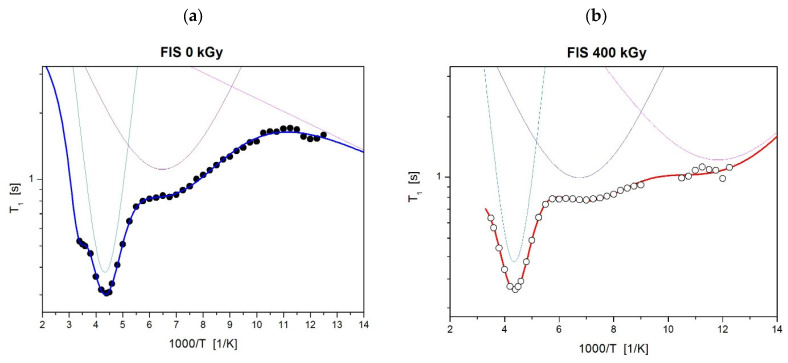
The temperature dependence of spin-relaxation time T_1_ for (**a**) FIS 0 kGy and (**b**) FIS 400 kGy. The solid lines are the best fits of Equations (3) and (4) to the experimental data.

By fitting Equations (3) and (4) to the experimental data presented in Figure 9, the activation parameters of molecular motions were calculated and are summarized in Table 7. The values of uncertainty for the estimated parameters were lower than 10%. In this context, τ_0_ represents the pre-exponential factor, E_a_ is the activation energy of molecular motions, and ΔM_2k_ denotes the reduction in the second moment in the ^1^H NMR spectra. Figure 10 depicts the optimal numerical fit to the experimental data using solid lines, with partial contributions represented by dotted lines.

Both for pure FIS and for the sample after irradiation with the dose of 400 kGy, a deep and sharp minimum at temperature T = 230 K is visible in Figure 9. The activation parameters obtained for this motion are nearly the same for both samples. It is assumed that the activation energy E_a_ about 20 kJ/mol may be related to oscillations of the phenyl ring. Analysis of the T_1_ relaxation times at lower temperatures reveals that for both FIS 0 kGy and 400 kGy samples, a broad minimum appears around 155 K, which may be attributed to the motion of the C=O group. Below 143 K, the T_1_ relaxation times for the FIS 400 kGy sample are significantly shortened, indicating changes in the structure and molecular dynamics of the irradiated drug.

Taking into account the lowest temperature range, it can be concluded that for FIS 0 kGy (Figure 10a), the third low-temperature motion, characterized by a relaxation time of τ_03_ = 2.8 × 10^−11^ s and a low activation energy of E_a__3_ = 1.1 kJ/mol, may be associated with hydrogen atom jumps in hydrogen bonds formed between molecules containing OH hydroxyl groups [34]. This motion is absent in FIS 400 kGy (Figure 10b), supporting the hypothesis that ionizing radiation disrupts hydrogen bonds between molecules, leading to their degradation. Figure 10b also shows a broad minimum at approximately 83 K, which is suggested to correspond to additional local group motion emerging in the irradiated sample. The analysis of NMR data confirms that irradiation induces changes in the molecular dynamics and structure of the drug.

## 4. Limitations and Future Perspective

Although this study provides encouraging insights into the anticancer potential of FIS and its nanoformulations, certain limitations must be acknowledged. The biological evaluation was limited to in vitro experiments on a single glioblastoma cell line (U-138 MG), which does not fully reflect the heterogeneity and complexity of glioma tumors in vivo. Furthermore, while the FIS-PLGA-NPs showed significantly enhanced cytotoxicity, the mechanisms of cellular uptake, intracellular drug release, and in vivo biodistribution remain unexplored. Additionally, long-term stability and potential immunogenicity of both free and encapsulated FIS were not evaluated, which are critical aspects for clinical translation.

In the present study, PLGA nanoparticles were intentionally developed in a non-coated form, which may limit BBB permeability when compared with surface-engineered or ligand-targeted systems. However, the primary objective of this work was to obtain FIS-loaded PLGA nanoparticles with high EE% and investigate their cytotoxicity in the in vitro glioblastoma model to verify the therapeutic potential of such a formulation before further functionalization. As widely documented in the literature, surface engineering approaches, such as surfactant coatings with polysorbate 80 or poloxamer 188 [35], and ligand functionalization with targeting moieties such as glutathione or phenylalanine [36], can significantly enhance active BBB transport via receptor-mediated mechanisms. In accordance with these findings, such modifications would be implemented in the next phase of formulation development, followed by comprehensive studies assessing drug release and biodistribution. Therefore, the present findings should not be interpreted as a final therapeutic solution, but as an initial step toward formulating an injectable fisetin delivery system, in which high drug entrapment serves as a critical starting point for future development.

## 5. Conclusions

This study highlights FIS as a promising candidate for glioblastoma therapy, demonstrating its ability to inhibit tumor cell proliferation through the induction of cell cycle arrest and apoptosis. The development of FIS-PLGA-NPs significantly improved the drug’s cytotoxic activity in vitro. Such formulation may enhance cellular uptake and allow for controlled release. Moreover, a comprehensive physicochemical assessment confirmed that FIS maintains structural and chemical stability after exposure to ionizing radiation, including the standard sterilization dose of 25 kGy, supporting the feasibility of using radiation sterilization as an effective method for obtaining a sterile active pharmaceutical ingredient. While higher doses induced transient molecular-level changes, no irreversible degradation was observed. Collectively, these findings support the feasibility of developing nanoparticle-based FIS formulation for glioblastoma treatment and provide a solid foundation for further preclinical investigation.

## Figures and Tables

**Table 1:** Formulation parameters for FIS-PLGA-NPs.

Sample	FIS:PLGA Ratio	PVA [%]	Sonication Time [min]
FIS-PLGA-NP1	1:20	1	2
FIS-PLGA-NP2	1:5		
FIS-PLGA-NP3	1:20	2	4
FIS-PLGA-NP4	1:5		
FIS-PLGA-NP5	1:20	3	6
FIS-PLGA-NP6	1:5		

**Table 2:** The EPR spectrometer working parameters.

Parameter	Value
Modulation frequency [kHz]	100,000
Center field [mT]	338
Sweep width [mT]	15
Sweep time [s]	60
Time constant [s]	0.008
Second modulation amplitude [G]	1
Radio Frequency power [mW]	0.291
Radio frequency [GHz]	9.460048
Temperature [K]	296.15

**Table 3:** The precision and accuracy of the HPLC methods of FIS determination after radiation.

Precision/Recovery				
Intra-Day				Inter-Day
Series I n = 9		Series II n = 9		Series I-II n = 18
Concentration [μg/mL]	RSD [%] Recovery [%]	Concentration [μg/mL]	RSD [%] Recovery [%]	RSD [%] Recovery [%]
20.06	1.15 96.87 ± 0.86	20.02	1.15 102.57 ± 0.91	3.14 99.72 ± 1.56

**Table 4:** Characterization of the studied FIS-Loaded PLGA NPs.

Sample	MPS ± SD [nm]	PDI ± SD	ZP ± SD [mV]	DL% ± SD [%]	EE% ± SD [%]
Formulation conditions I: 1% PVA; 2 min. of emulsification					
FIS-PLGA-NP1 *	660 ± 80	0.45 ± 0.03	−13.60 ± 0.44	0.89 ± 0.20	18.69 ± 4.16
FIS-PLGA-NP2 **	560 ± 21	0.49 ± 0.03	−11.37 ± 0.15	13.05 ± 1.80	78.30 ± 10.78
Formulation conditions II: 2% PVA; 4 min. of emulsification					
FIS-PLGA-NP3 *	323 ± 2	0.20 ± 0.01	−12.27 ± 0.81	0.61 ± 0.01	12.71 ± 0.15
FIS-PLGA-NP4 **	330 ± 6	0.25 ± 0.03	−7.18 ± 1.19	13.93 ± 0.07	83.58 ± 0.42
Formulation conditions II: 3% PVA; 6 min. of emulsification					
FIS-PLGA-NP5 *	380 ± 7	0.23 ± 0.01	−10.00 ± 0.09	0.57 ± 0.01	11.87 ± 0.15
FIS-PLGA-NP6 **	300 ± 4	0.18 ± 0.01	−6.92 ± 0.90	11.81 ± 0.28	70.86 ± 1.70

Fisetin (FIS)-to-PLGA ratio: * 1:20; ** 1:5, MPS—mean particles size, PDI—polydispersity index, ZP—zeta potential, DL%—drug loading, EE%—entrapment efficiency, n = 3.

**Table 5:** Results of EPR analysis.

Parameter	0 kGy	25 kGy	100 kGy	400 kGy
g-factor	2.0028	2.0031	2.0032	2.0031
dH [mT]	0.82	0.8	0.83	0.82
Signal amplitude	63.1	189.6	264.6	322.0
Signal amplitude after 2 months	30.5	74.2	105.6	154.8
Line center [mT]	337.46	337.38	337.41	337.52

**Table 6:** Determination of FIS content in samples before and after radiation.

Dose of Radiation	Average Amount [%]
0 kGy	99.72 ± 1.56 *
25 kGy	98.20 ± 2.38 **
100 kGy	97.70 ± 1.14 **
400 kGy	97.13 ± 1.37 **

The results are presented as mean value ± value of absolute error from nine measurements, * expressed as a percentage of theoretical content or ** relative to the non-irradiated substance.

**Table 7:** Activation parameters of the internal motions obtained for FIS before and after irradiation.

Sample	Internal Motion		
FIS 0 kGy	oscillations of the ring	motion of local group (C=O)	proton jump in hydrogen bonds
	τ_02_ = 0.9$\;\cdot 10^{-13}(s)$ E_a2_ = 20.4 (kJ/mol) $∆M_{2}$ = 0.6 (10^−8^T^2^)	τ_03_ = 6.7$\;\cdot 10^{-11}(s)$ E_a3_ = 5.2 (kJ/mol) $∆M_{3}$ = 0.2 (10^−8^T^2^)	τ_03_ = 2.8$\;\cdot 10^{-11}(s)$ E_a3_ = 1.1 (kJ/mol) $∆M_{3}$ = 1.6 (10^−8^T^2^)
FIS 400 kGy	oscillations of the ring	motion of local group (C=O)	additional local motion in the low temperature
	τ_02_ = 0.9$\;\cdot 10^{-13}(s)$ E_a2_ = 20.3 (kJ/mol) $∆M_{2}$ = 0.5 (10^−8^T^2^)	τ_03_ = 6.4$\;\cdot 10^{-11}(s)$ E_a3_ = 5.1 (kJ/mol) $∆M_{3}$ = 0.2 (10^−8^T^2^)	τ_03_ = 3.6$\;\cdot 10^{-11}(s)$ E_a3_ = 3.3 (kJ/mol) $∆M_{3}$ = 0.2 (10^−8^T^2^)

## Data Availability

The raw data supporting the conclusions of this article will be made available by the authors on request.
